# Signal Injection as a Fault Detection Technique

**DOI:** 10.3390/s110303356

**Published:** 2011-03-21

**Authors:** Jordi Cusidó, Luis Romeral, Juan Antonio Ortega, Antoni Garcia, Jordi Riba

**Affiliations:** MCIA Research Group, Universitat Politècnica de Catalunya, C. Colom 1, Terrassa, 08222 Catalunya, Spain; E-Mails: romeral@eel.upc.edu (L.R.); ortegar@eel.upc.edu (J.A.O.); garciae@ee.upc.edu (A.G.); riba@ee.upc.edu (J.R.)

**Keywords:** fault detection, induction motor, electrical drives

## Abstract

Double frequency tests are used for evaluating stator windings and analyzing the temperature. Likewise, signal injection on induction machines is used on sensorless motor control fields to find out the rotor position. Motor Current Signature Analysis (MCSA), which focuses on the spectral analysis of stator current, is the most widely used method for identifying faults in induction motors. Motor faults such as broken rotor bars, bearing damage and eccentricity of the rotor axis can be detected. However, the method presents some problems at low speed and low torque, mainly due to the proximity between the frequencies to be detected and the small amplitude of the resulting harmonics. This paper proposes the injection of an additional voltage into the machine being tested at a frequency different from the fundamental one, and then studying the resulting harmonics around the new frequencies appearing due to the composition between injected and main frequencies.

## 1. Introduction

**Figure 1.**
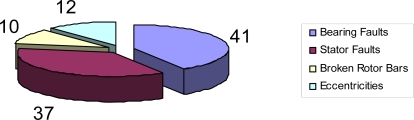
Statistical distribution of motor faults.

The history of fault diagnosis and protection is as old as machines themselves. The manufacturers and users of electrical machines initially relied on simple protection against problems like overcurrent, overvoltage, earth-faults, *etc.*, to ensure safe and reliable operation. However, as the tasks performed by these machines became more complex, improvements were also sought in the field of fault diagnosis. It has now become very important to be able to diagnose faults at their very inception, as unscheduled machine downtime can upset deadlines and cause enormous financial losses. The major faults of electrical machines can broadly be classified as follows:

***Electrical Faults*:**
1. stator faults resulting in the opening or shorting of one or more stator windings;
2. abnormal connection of the stator windings;

***Mechanical Faults*:**
3. broken rotor bars or rotor end-rings;
4. static and/or dynamic air-gap irregularities;
5. bent shaft (similar to dynamic eccentricity) which can result in frictions between the rotor and the stator, causing serious damage to the stator core and the windings;
6. bearing and gearbox failures.

and the frequency at which different kinds of fault typically occur is shown in Figure 1:

Operating a machine under faults generates at least one of the following symptoms:

1. unbalanced air-gap voltages and line currents;
2. increased torque pulsations;
3. decreased average torque;
4. increase in losses and decrease in efficiency;
5. excessive heating.

Many diagnostic methods have been developed for detecting such fault-related signals. These methods come from different types and areas of science and technology, and can be summarized as follows [1–4]:

1. Electromagnetic field monitoring by means of search coils, and coils placed around motor shafts (axial flux-related detection). This is associated with the capacity for capturing the presence of magnetic fields around an IM. Field evaluation must provide information about motor-operation states as proposed by Zidat *et al.* [4], but this is an intrusive proposal.
2. Temperature measurements: temperature is a typical second-order effect in operation conditions. Induction motors typically have an operational temperature range, defined in the motor nameplate, and associated with tests performed. Any fault-operation condition shows a temperature increment. By performing a temperature analysis the first approach to identifying fault conditions could be made.
3. Infrared recognition: this is used to evaluate the material state, especially for bearings. This cannot be performed in an online system.
4. Radio frequency (RF) emissions monitoring: radio frequency is a second-order effect of a fault condition, which is currently used for gearbox diagnosis.
5. Vibration monitoring: this is the typical method for fault diagnosis in industrial applications; it achieves good results for bearing analysis, but presents some deficiencies with electrical and rotor faults [5,6].
6. Chemical analysis: this is used to analyze bearing grease; it is used only with large motors and not with the more typical small ones.
7. Acoustic noise measurement: this is a new trend in the field of gearbox failure detection.
8. Motor current signature analysis (MCSA), which is explained further below.
9. Model-based artificial intelligence and neural-network-based techniques. These are new approaches which combine multi-modal data acquisition and advanced signal-processing techniques introduced by Nandi *et al.* [7].

The present work is not an attempt to develop fault diagnosis for all recognized methods, but instead focuses on the analysis of the motor current signature analysis (MCSA) technique. This technique has been chosen for its recognition as an industrial standard and as a non-invasive technique. The basis of this technique is widely known and has been introduced by several authors. Among them, Toliyat *et al.* [7,8], Benbouzid *et al.* [9,10], and Thomson [11,12] are the most relevant in the field, although many others [13–20] have also contributed to scientific advances in the area.

These publications introduce the basis of MCSA operations, which are also the basis of this research project. Many of the authors deal with mechanical faults, especially with the effects of broken rotor bars and eccentricities. Thomson, though, focuses on stator fault diagnosis and presents good results and arguments. These works are a good introduction to MCSA condition-monitoring techniques and give a clear overview of the analysis of faults in induction machines for steady-state operations.

Power supply in induction machines creates a rotating magnetic field on the armature. The rotating magnetic field induces rotor voltages and currents at slip frequencies, and this generates an effective three-phase magnetic field rotating at slip frequency with respect to the rotor. Two different cases appear:

- Symmetrical cage winding ⇒ only forward rotating field is produced.
- Asymmetric rotor ⇒ a backward rotating field will result at slip frequency with respect to the rotor.

This backward rotating field induces a voltage in the stator at the corresponding frequency, and generates a related current which modifies the stator-current spectra. Different rotating fields appear with different faults in the induction machine, such as air-gap eccentricity, broken rotor bars, bearing damage and short circuits in the stator windings. The current frequencies associated with rotating fields are expressed by Equations (1–4):

- Air-gap eccentricity fault [9,10]
  (1) $$f_{\mathrm{ecc}}=f_{s}\left[1\pm m\left(\frac{1-s}{p}\right)\right]$$
  where m = 1,2,3,… is a positive integer, *p* is the number of pole pairs, *s* is the per-unit slip, and *f_s_* is the electrical supply frequency.
- Broken rotor bars [7,8]
  (2) $$f_{\mathrm{bdb}}=f_{s}\left[l\;\left(\frac{1-s}{p}\right)\pm s\right]$$
  where l/p = 1,5,7,11,13,…are the characteristic values of the motor.
- Bearing damage [9,10]
  (3) $$\begin{array}{l} f_{\mathrm{bng}}=\left|f_{s}\pm {\mathrm{mf}}_{i,o}\right|\\ f_{i,o}=\frac{n_{b}}{2}f_{r}\left[1\pm \frac{\mathrm{bd}}{\mathrm{pd}}\cos\;\beta \right] \end{array}$$

where *n_b_* is the number of bearing balls, *f*_*i*,0_ are the characteristic vibration frequencies, *f_r_* is the speed of the mechanical rotor in Hz, *b_d_* is the ball diameter, *p_d_* is the bearing pitch diameter, and *β* is the contact angle of the balls with the races.

Equation (3) shows the frequency vibration of a motor with a broken bearing; however these harmonics cannot be easily appreciated on currents. In fact, the case of bearing damage causes rotor eccentricity, and furthermore the appearance of eccentricity on the rotor or even on the load will cause further bearing damage. For this reason, we can also use Equation (1) to detect bearing problems.

(d) Shorted turns

d. (1) medium frequencies
  (4) $$f_{\mathrm{sth}}=f_{s}\left[1\pm {\mathrm{mZ}}_{2}\left(\frac{1-s}{p}\right)\right]$$
d. (2) low frequencies
  (5) $$f_{\mathrm{stl}}=f_{s}\left[\frac{m}{p}\left(1-s\right)\pm k\right]$$

where *Z_2_* is the number of rotor slots or rotor bars and k = 0,1,3,5,...

Expression (4) shows the components produced by shorted turns in the air-gap flux waveform, and hence the stator currents as a function of rotor slots, around the medium-order harmonics, while Expression (5) shows the harmonics produced by the fault around the base frequency *f_s_*. However, frequencies shown by (5) also appear in the case of any rotor unbalance, including eccentricities, rotor misalignment, *etc.* Therefore, (4) is frequently used to detect the fault, and (5) is used to assure the origin in shorted turns in the stator winding.

**Figure 2.**
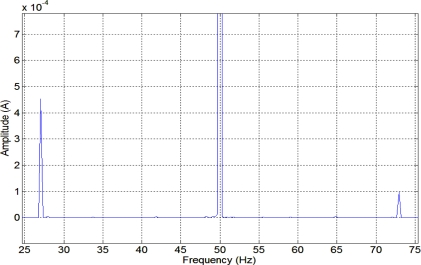
Stator current spectrum of an induction motor with high eccentricity at nominal load.

**Figure 3.**
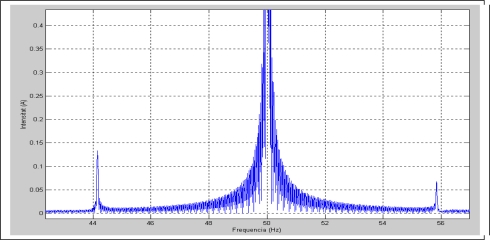
Stator current spectrum of an induction motor with eight broken bars.

Figure 2 depicts the stator current spectrum of the induction machine. The harmonic frequencies produced by the fault are clearly shown at 25 Hz, 75 Hz, 125 Hz and 175 Hz, as expected from (1). Figure 3 depicts the stator current spectrum for a constant load of the induction motor with broken bars, one-sixth of the total in this case. As expected, an important harmonic appears in the lower sideband of the main frequency.

The effects of electrical faults on induction machines are clearly introduced by Thomson [11,12], while some other authors [17,18] work with current monitoring without spectral analysis. In the case of stator faults, spectral analysis may not be needed. However, it is worth considering if we are aiming for a global solution for the fault diagnosis of induction machines.

Having acquired this knowledge about motor behavior under healthy and faulty conditions and its relation to the distribution of harmonics, deeper studies for improving fault detection could be carried out. As previously described, MCSA is a good fault-detection technique, which has achieved good results in numerous cases. However, its drawbacks do not allow a global solution for an online condition-monitoring technique or the development of diagnostic tools.

The main drawbacks are related to the fact that induction machines do not operate with a constant low torque and at a constant speed. Induction machines have become increasingly popular, especially since inverter drives appeared on the market. Nowadays, squirrel-cage motors cover most industrial and domestic applications and are the most important way of converting electrical energy to mechanical energy. These motors work with different kinds of applications with constant and variable loads, and at constant and variable speeds. Moreover, inverters introduce additional drawbacks in motors, such as common mode voltages, *dv/dt*, and additional harmonics. A global solution is needed and induction machines in different operating positions should be studied further. The main purpose of this work is to develop new fault-detection techniques for any operating condition.

Different solutions have been introduced in order to minimize the problems related to proper fault identification under non-standard load conditions. Some are based on flux measurement in the stator teeth [21], or by performing higher-order statistical analyses [22].

Important trends in fault detection are the injection of additional frequency tests and the development of new tools based on improved signal-processing techniques, such as the Wavelet Transform or dq0 conversions. The first introduction of signal injection can be found in the EN 61986-2002 standard used for motor insulation evaluation. In 1998 Ho and Cheng [23] introduced the low-frequency signal injection on faulty machines, which proved to be a good approach with some very interesting results. However this is far from being a full solution, since it fails to take into account the effects of the signal injection, such as the composition between injected and fundamental harmonics.

In a paper published in 2004 [24], Henao, Capolino *et al*. developed the idea of mechanical fault detection by injecting different excitation signals, such as a discrete interval binary sequence (DIBS) and multisine, with the intention of exciting faulty modes with the low frequency resolution and analyzing the stator current and the stray flux measured by an external flux sensor. This work, regardless of being based on the analysis of stray flux, offers an interesting approach to faulty motor behavior excited by different injected signals.

Two articles published in 2003 and 2004 [25,26], by Briz and co-workers, use high-frequency injection as a method of detecting winding faults in the first paper, and rotor faults in the second. The measurement of the negative-sequence carrier-signal currents, using low-magnitude high-frequency voltage superimposed by the fundamental excitation voltage, was shown to reliably detect faults in the stator windings and the rotor cage (broken rotor bars) at their incipient stage, regardless of the working condition of the machine. This is also an interesting approach, which we have considered in our work, although the effect of signal compositions has been not taken into account. These works [24–26] show the injection of additional signals as a good technique for fault detection. However, the effects of frequency composition and behavior under double frequency (injected plus fundamental) are not clearly shown. These subjects are developed, and supported by theoretical analysis, simulations and experimental results. As already introduced, injection can be a good method of analyzing motors driven by power inverters, which could implement a diagnostic routine.

## 2. Proposed Approach

Due to the effects of induction we expect to see both the main frequency and the auxiliary frequency injected in the spectrum. However, as a contribution of the magnetic nucleus and iron hysteresis, and also due to the general non-linearity of the induction motor, additional compositions appear, defined by the following equation:

(6) $$f_{c}=n\cdot f_{s}+m\cdot f_{i}$$

where *n* = *m* = ...−2, −1, 0, 1, 2, …, and *f_c_* > 0.

It is possible to determine the effect of broken rotor bars in the motor’s current spectrum by studying the flux composition in the stator and the mechanical composition of frequencies as a speed composition. In the stator there are different magnetic fields due to the different signal injections. If different fields are considered as different wheels moving around themselves with different angular speeds, relative speeds between them will become evident.

Moreover, if the rotor is taken into consideration, it will be easy to define the different relative speeds between the rotor and all the stator fields. The relation equations between rotor currents and stator currents in an induction machine establish the former as an image of the latter. For instance, if the rotor has salients such as broken bars, these will have an effect on stator currents as images. In an ideal induction machine, all the different current distributions will be sine-shaped like the fields, but there are many effects that cause non-idealities. In addition, any change in the air-gap flux distribution can be seen as a non-ideal effect and will cause some marks in the current spectrum, as well as around the different injected signals.

To determine these different marks, it is necessary to study the composition of the different frequencies, the different magnetic fields induced in the machine, and the relative speed between them. In (7) we shall consider the rotational speed of the motor *f_r_*:

(7) $$f_{r}=f_{s}\frac{\left(1-s\right)}{p}$$

Broken bars or rings, fractures in the squirrel cage, and other faults in the rotor will lead to pulsating fields, which can be seen as two rotational fields rotating at slip frequency:

(8) $$f_{\mathrm{rotational}}=\pm {\mathrm{sf}}_{s}$$

From the point of view of stator windings, the backward component of the rotor bar failure is seen at frequency (−*sf_s_* *+ l f_r_*), where l is the function of pole pairs. This means:

(9) $$f_{\mathrm{bb\_back}}=f_{s}\left[l\left(\frac{1-s}{p}\right)-s\right]$$

corresponding to the broken rotor bars frequencies in the left sideband. Note that the forward component of the rotating field in the rotor does not produce any new harmonic in the stator spectrum.

If a three-phase test signal is injected in the stator at frequency *f_i_*, new rotational components are again produced in the rotor at frequencies *± (f_i_* - *f_c_* - *f_r_)*, where *f_c_* are new composed frequencies such as (6). The rotating image fields produced in the stator are seen at *± (f_c_* − *f_r_) ± f_r_*. A general expression can be obtained that includes all the harmonics of the main and injected frequencies:

(10) $$f_{\mathrm{bb\_back\_inj}}=\pm \;n\;f_{s}\pm m\;f_{i}-2\;j\;{\mathrm{sf}}_{s}$$

where *j* = 1, 3, 4, 6, ..

The faulty frequency components that appear in the stator are not only due to the injected signals, but also to the composed frequencies specified by (6). Harmonic components produced by the failure in the rotor are expected to be found around the composed and corresponding harmonics of these new frequencies.

The motor could be considered as a low-pass filter with a pole frequency of 400 Hz. Since different injected frequencies will produce different compositions, the injected signals should be chosen to obtain composed frequencies between four times *f_s_* and 400 Hz. In this way, the optimum bandwidth is windowed to analyze the stator current spectrum without affecting the motor operation.

The main (and sometimes the only) solutiion when a motor fails is to repair it or to replace it. On the contrary, the approach presented allows setting up permanent supervision and predictive maintenance actions on the motor and the associated chain. The way to implement the frequency injection test is as simple as injecting frequency components from the inverter source and analyzing frequency bands around the new harmonics appearing on the stator current.

## 3. Simulation Analysis

The objective of the preceding modeling was to estimate the impedance variation due to faults. The typical parametric model for induction machines is presented in Equations (11), (12) and (13). They express the voltage relationship between rotor and stator (11), torque (12), and speed and rotor position Equations (13).

(11) $$\left[\begin{array}{l} \left[V_{s}\right]\\ \left[V_{r}\right] \end{array}\right]=\left[\begin{array}{cc} \left[R_{s}\right] & \left[0\right]\\ \left[0\right] & \left[R_{r}\right] \end{array}\right]\left[\begin{array}{l} I_{s}\\ I_{r} \end{array}\right]+\frac{d}{\mathrm{dt}}\left[\begin{array}{cc} \left[L_{\mathrm{ss}}\;\left(\theta \right)\right] & \left[L_{\mathrm{sr}}\;\left(\theta \right)\right]\\ \left[L_{\mathrm{rs}}\;\left(\theta \right)\right] & \left[L_{\mathrm{rr}}\;\left(\theta \right)\right] \end{array}\right]\left[\begin{array}{l} I_{s}\\ I_{r} \end{array}\right]$$

(12) $$T_{\mathrm{elec}}\;(t)={\left[I_{s}\right]}^{t}\;\frac{d}{d\theta }\;\left[L_{\mathrm{sr}}\;\left(\theta \right)\right]\left[I_{r}\right]$$

(13) $$\frac{{d\omega }_{m}}{\mathrm{dt}}=\frac{1}{J}\left(T_{\mathrm{elec}}\;-\;T_{\mathrm{mec}}\;\left(\theta \right)\right)\;\;\;;\;\frac{d\theta }{\mathrm{dt}}=\omega_{m}$$

### 3.1. Rotor Misalignment

Rotor misalignment can be expressed as a variation on mutual inductances between rotor and stator windings. This variation pulses at the frequency *s f_s_* referring to stator fields.

This means a variation on mutual inductances of:

(14) $$\begin{array}{l} k_{1}\;=\left|k\;\text{cos}\;\;\left(2\;\cdot \;\pi \;\cdot \;s\;\cdot \;f_{s}\right)\right|\\ k_{2}\;=\left|k\;\text{cos}\;\;\left(2\;\cdot \;\pi \;\cdot \;s\;\cdot \;f_{s}\;+\;\frac{2\pi }{3}\right)\right|\\ k_{3}\;=\left|k\;\text{cos}\;\;\left(2\;\cdot \;\pi \;\cdot s\;\cdot f_{s}\;-\;\frac{2\pi }{3}\right)\right| \end{array}$$

Giving a final expression of inductances:

(15) $$\left[\begin{array}{ll} \left(\begin{array}{ccc} L_{\mathrm{SA}} & M_{S_{\mathrm{AB}}} & M_{S_{\mathrm{AC}}}\\ M_{S_{\mathrm{BA}}} & L_{\mathrm{SB}} & M_{S_{\mathrm{BC}}}\\ M_{S_{\mathrm{CA}}} & M_{S_{\mathrm{CB}}} & L_{\mathrm{SB}} \end{array}\right) & \left(\begin{array}{lll} M_{{\mathrm{SR}}_{\mathrm{AA}}}\;\left(1+\;k_{1}\right) & M_{{\mathrm{SR}}_{\mathrm{AB}}}\;\left(1+\;k_{2}\right) & M_{{\mathrm{SR}}_{\mathrm{AC}}}\;\left(1+\;k_{3}\right)\\ M_{{\mathrm{SR}}_{\mathrm{BA}}}\;\left(1+\;k_{3}\right) & M_{{\mathrm{SR}}_{\mathrm{BB}}}\;\left(1+\;k_{1}\right) & M_{{\mathrm{SR}}_{\mathrm{BC}}}\;\left(1+\;k_{2}\right)\\ M_{{\mathrm{SR}}_{\mathrm{CA}}}\;\left(1+\;k_{2}\right) & M_{{\mathrm{SR}}_{\mathrm{CB}}}\;\left(1+\;k_{3}\right) & M_{{\mathrm{SR}}_{\mathrm{CC}}}\;\left(1+\;k_{1}\right) \end{array}\right)\\ \left(\begin{array}{ccc} M_{{\mathrm{RS}}_{\mathrm{AA}}}\;\left(1+\;k_{1}\right) & M_{{\mathrm{RS}}_{\mathrm{AB}}}\;\left(1+\;k_{2}\right) & M_{{\mathrm{RS}}_{\mathrm{AC}}}\;\left(1+\;k_{3}\right)\\ M_{{\mathrm{RS}}_{\mathrm{BA}}}\;\left(1+\;k_{3}\right) & M_{{\mathrm{RS}}_{\mathrm{BB}}}\;\left(1+\;k_{1}\right) & M_{{\mathrm{RS}}_{\mathrm{BC}}}\;\left(1+\;k_{2}\right)\\ M_{{\mathrm{RS}}_{\mathrm{CA}}}\;\left(1+\;k_{2}\right) & M_{{\mathrm{RS}}_{\mathrm{CB}}}\;\left(1+\;k_{3}\right) & M_{{\mathrm{RS}}_{\mathrm{CC}}}\;\left(1+\;k_{1}\right) \end{array}\right) & \left(\begin{array}{ccc} L_{\mathrm{RA}} & M_{R_{\mathrm{AB}}} & M_{R_{\mathrm{AC}}}\\ M_{R_{\mathrm{BA}}} & L_{\mathrm{RB}} & M_{R_{\mathrm{BC}}}\\ M_{R_{\mathrm{CA}}} & M_{R_{\mathrm{CB}}} & L_{\mathrm{SB}} \end{array}\right) \end{array}\right]$$

### 3.2. Broken Rotor Bars

The incidence of broken rotor bars (BRB) must appear principally as a variation on rotor resistances. In fact, BRB incidences produce changes in both rotor resistances and inductances. However, for broken rotor bars, variations of resistance in one rotor phase allow proper results to be achieved. The actual degree of error depends on the number of bars the rotor cage has, the number of contiguous broken bars, and the damage in the degrading bar(s). Since Rra is the equivalent resistance of parallel n/3 rotor bars, if all but one rotor bar are healthy then the relationship can be obtained by the following Equation (16):

(16) Rra′=α ⋅ Rra=Rrai(n3−1) ⋅ k ⋅ RraiRrai(n3−1)+k ⋅ Rrai=k1+(n3−1) ⋅RraiRra=Rrain3α=Rra′Rra=k ⋅ n3 + (n−3) ⋅ k

For example, for a 12 bar in a rotor cage, an increase in Rra by a factor of 1.328 (*i.e.*, α = 1.328 above and R’ra = 1.328·Rra) would mean that the resistance of one rotor bar had increased by a factor of 83 (k = 83), if the other bars were not damaged. If there are m contiguous broken bars and two bars next to them with the same damage k, then the R’ra/Rra relationship would be:

(17) Rra′=α ⋅ Rra=Rrai(n3−m−2) ⋅ k ⋅ Rrai2Rrai(n3−m−2)+k ⋅ Rrai2=k2+(n3−m−2) ⋅ k ⋅ RraiRra=Rrain3α=Rra′Rra=k ⋅ n6+(n−3m−6) ⋅ k

Furthermore, resistance exchange would be an inductance variation happening on misalignment, rather than a mutual inductance variation appearing as variations in the self phase inductance L, due to the variation in the number of rotor bars and a variation in mutual inductance, M (between the rotor and stator) due to the reluctance exchange. The variations on R, L and M would pulse at rotor relative speeds, and referring to stator rotating flux, this pulsation is *s f_s_*, giving:

In the case of rotor resistance:

(18) $$\begin{array}{l} \alpha_{1}=\left|\alpha \;\text{cos}\;\;\left(2\;\cdot \;\pi \;\cdot \;s\;\cdot \;f_{s}\right)\right|\\ \alpha_{2}=\left|\alpha \;\text{cos}\;\;\left(2\;\cdot \;\pi \;\cdot \;s\;\cdot \;f_{s}\;+\;\frac{2\pi }{3}\right)\right|\\ \alpha_{3}=\left|\alpha \;\text{cos}\;\;\left(2\;\cdot \;\pi \;\cdot \;s\;\cdot \;f_{s}\;-\;\frac{2\pi }{3}\right)\right| \end{array}$$

In the case of rotor self-inductance:

(19) $$\begin{array}{l} \kappa_{1}=\left|\kappa \;\text{cos}\;\;\left(2\;\cdot \;\pi \;\cdot \;s\;\cdot \;f_{s}\right)\right|\\ \kappa_{2}=\left|\kappa \;\text{cos}\;\;\left(2\;\cdot \;\pi \;\cdot \;s\;\cdot \;f_{s}\;+\;\frac{2\pi }{3}\right)\right|\\ \kappa_{3}=\left|\kappa \;\text{cos}\;\;\left(2\;\cdot \;\pi \;\cdot \;s\;\cdot \;f_{s}\;-\;\frac{2\pi }{3}\right)\right| \end{array}$$

For the *κ* version, an equivalent equation can be used as given for *α* in the rotor resistance case, depending on the number of rotor bars n, and degree of damage on rotor bars k:

(20) $$\kappa =\frac{L_{\mathrm{ra}}^{\prime }}{L_{\mathrm{ra}}}=\frac{k\;\cdot \;n}{3+\left(n-3\right)\;\cdot \;k}$$

Mutual inductance must fulfill the same expression (14) as in the case of eccentricity.

These variations will give the equation substitutions on fundamental motor equations, which for the case of broken rotor bars gives:

(21) $$\left[R_{r}\right]=\left[\begin{array}{ccc} R_{\mathrm{ra}}\;\left(1+\alpha_{1}\right) & 0 & 0\\ 0 & R_{\mathrm{rb}}\;\left(1+\alpha_{2}\right) & 0\\ 0 & 0 & R_{\mathrm{rc}}\;\left(1+\alpha_{3}\right) \end{array}\right]$$

(22) $$\left[\begin{array}{ll} \left(\begin{array}{ccc} L_{\mathrm{SA}} & M_{S_{\mathrm{AB}}} & M_{S_{\mathrm{AC}}}\\ M_{S_{\mathrm{BA}}} & L_{\mathrm{SB}} & M_{S_{\mathrm{BC}}}\\ M_{S_{\mathrm{CA}}} & M_{S_{\mathrm{CB}}} & L_{\mathrm{SB}} \end{array}\right) & \left(\begin{array}{ccc} M_{{\mathrm{SR}}_{\mathrm{AA}}} & M_{{\mathrm{SR}}_{\mathrm{AB}}} & M_{{\mathrm{SR}}_{\mathrm{AC}}}\\ M_{{\mathrm{SR}}_{\mathrm{BA}}} & M_{{\mathrm{SR}}_{\mathrm{BB}}} & M_{{\mathrm{SR}}_{\mathrm{BC}}}\\ M_{{\mathrm{SR}}_{\mathrm{CA}}} & M_{{\mathrm{SR}}_{\mathrm{CB}}} & M_{{\mathrm{SR}}_{\mathrm{CC}}} \end{array}\right)\\ \left(\begin{array}{ccc} M_{{\mathrm{RS}}_{\mathrm{AA}}} & M_{{\mathrm{RS}}_{\mathrm{AB}}} & M_{{\mathrm{RS}}_{\mathrm{AC}}}\\ M_{{\mathrm{RS}}_{\mathrm{BA}}} & M_{{\mathrm{RS}}_{\mathrm{BB}}} & M_{{\mathrm{RS}}_{\mathrm{BC}}}\\ M_{{\mathrm{RS}}_{\mathrm{CA}}} & M_{{\mathrm{RS}}_{\mathrm{CB}}} & M_{{\mathrm{RS}}_{\mathrm{CC}}} \end{array}\right) & \left(\begin{array}{ccc} L_{\mathrm{RA}}\left(1+\kappa_{1}\right) & M_{R_{\mathrm{AB}}} & M_{R_{\mathrm{AC}}}\\ M_{R_{\mathrm{BA}}} & L_{\mathrm{RB}}\left(1+\kappa_{2}\right) & M_{R_{\mathrm{BC}}}\\ M_{R_{\mathrm{CA}}} & M_{R_{\mathrm{CB}}} & L_{\mathrm{SB}}\left(1+\kappa_{3}\right) \end{array}\right) \end{array}\right]$$

### 3.3. Simulink Motor Model Implementation

**Figure 4.**
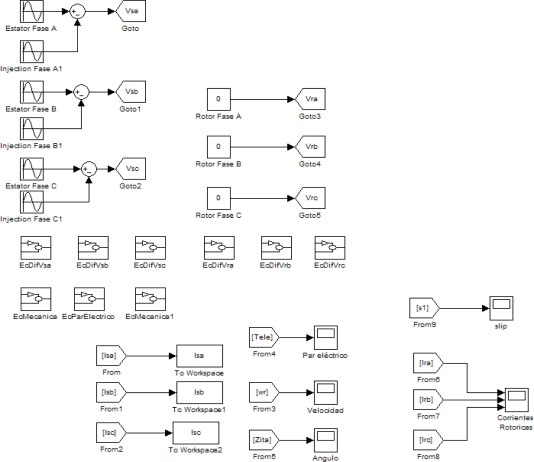
Implemented injections on the parametric model.

The parametric equation system just presented has been implemented on Simulink, with the different blocks containing differential equations for stator and rotor phases, torque and differential speed equations. In the differential equations for stator and rotor phases variable parameters have been introduced, which represent the fault condition. Three additional blocks have been added to the main model developed in Section 3 to introduce the additional frequency on the stator supply. The following Figure 4 shows the expected harmonic composition on stator currents due to the injection, and the appearance of the faulty harmonic at the frequency test and the additional composed harmonics.

**Figure 5.**
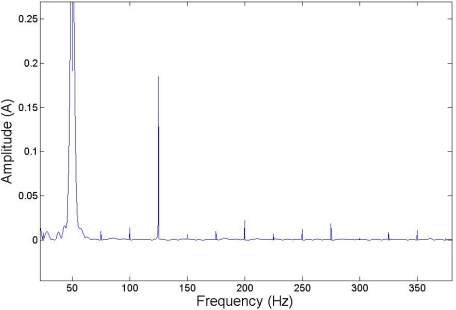
Injection of 125 Hz with no load; injected and composed harmonic distribution.

The composed frequencies appear only in the case of motor misalignment, increasing in amplitude with the increment of the fault condition. Figure 5 shows the expected harmonic distribution.

**Figure 6.**
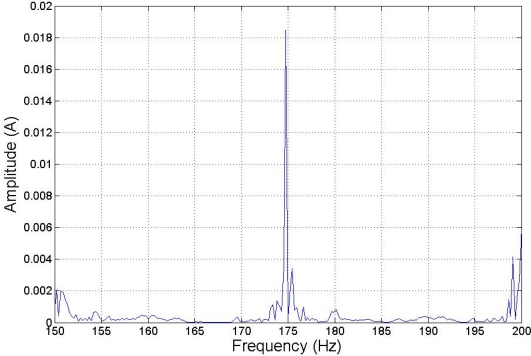
Detail of 175 Hz for 125 Hz injected frequency with low torque. This shows a BRB fault condition.

**Figure 7.**
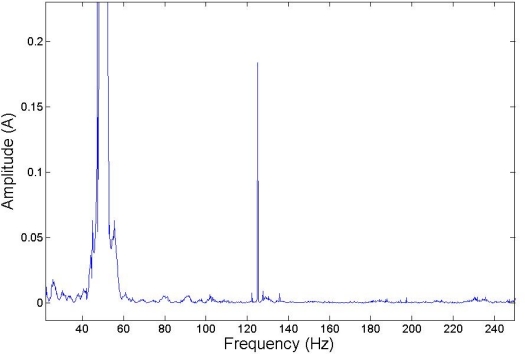
125 Hz Injected frequency test, low load.

**Figure 8.**
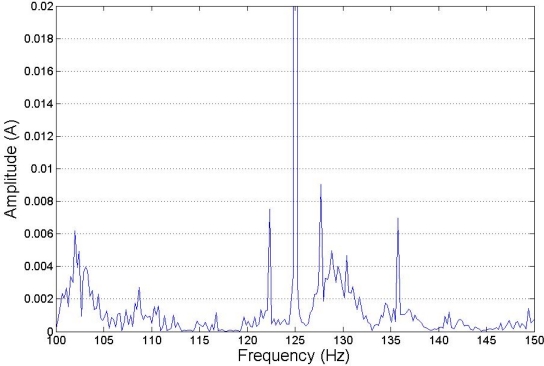
Injection of 125 Hz, low load. Detail.

Figures 6, 7 and 8 show how harmonics appear due to the fault condition around the injected and composed harmonics.

**Figure 9.**
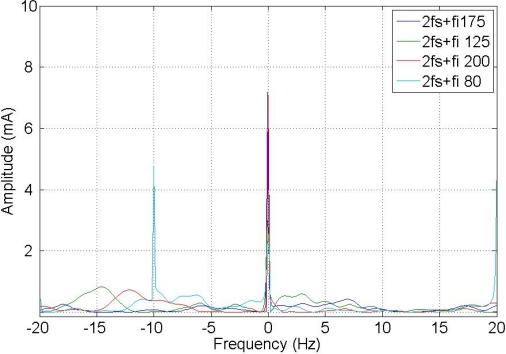
Composite frequencies 2 Fs + Fi.

**Figure 10.**
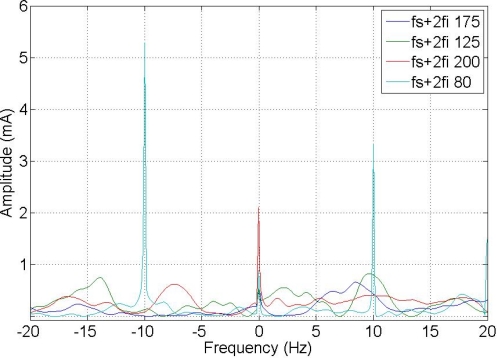
Composite frequencies Fs + 2 Fi.

Figures 9 and 10 show a comparison between different composite frequencies; composite frequencies appear only in the case of a fault condition, which implies a good fault-estimation parameter for a motor operating with no load.

**Figure 11.**
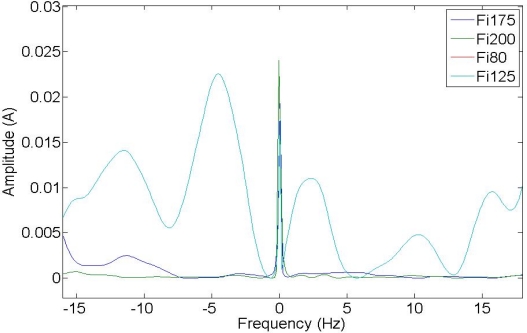
Composed frequencies 2 Fs–Fi.

Low-frequency composed harmonics cause torque oscillations, which are confusing for simulation results. Figure 11 shows frequency-composed harmonics at low frequencies, lower than the frequency supply. The variation in amplitude in some harmonics can be appreciated, due to the fault condition and torque oscillations during startup. These harmonics may hence be used to get good results in fault detection.

### 3.4. Influence of Injected Currents

**Figure 12.**
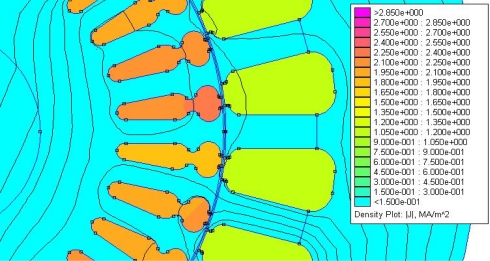
Flux density for 50 Hz frequency.

**Figure 13.**
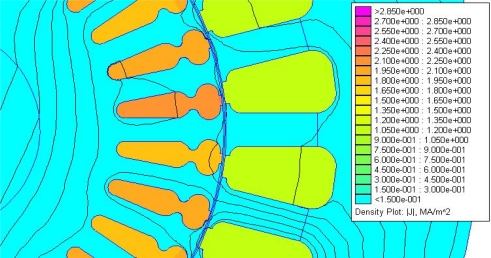
Flux density for 200 Hz frequency.

To consider the effect of saturation on the rotor sheet, the induced field has been simulated by means of FEM software. Different injected frequency tests will produce different effects on the motor; several papers [8] introduce us to the injection theories for sensorless control motors. These references talk about the motor as a band-pass. In order to ensure this, it is possible to simulate the flux density of current and field on the stator and squirrel cage, using a simulator properly, introducing rotor and stator design and introducing the frequency test found in Figure 12 (current flow density for 50 Hz frequency) and in Figure 13 (current flow density for 200 Hz frequency) for the same voltage amplitude.

Having a look at the last two figures we can see that for the 200 Hz frequency test there is a bigger current density, which confirms the idea that the motor could be considered as a band-pass with 200 Hz of central frequency of the band. In order to do this, we will try to inject our frequency test as close as possible to 200 Hz.

Regarding the effect of saturation, the FEM analysis shows the flux distribution on the motor sheet to be similar for the injected frequencies under analysis. Therefore, injecting a low current of frequency test does not produce saturation on the motor sheet.

## 4. Experimental Procedure

### 4.1. Test Rig Experimental Setup

A three-phase, 1.1 kW, 380 V and 2.6 A, 50 Hz, 1,410 rpm, four-pole induction motor was used in this study. First of all, its healthy performance was analyzed and, afterwards, one-sixth of the rotor bars were damaged. The current has been measured by an A622 Tektronix current probe, 100 Ampere AC/DC. The current ranges are 0/100 mV/A, and the typical DC accuracy is ±3% ± 50 mA at 100 mV/A (50 mA to a 10 A peak). The frequency range goes from DC to 100 kHz (−3 dB).

### 4.2. Signal Acquisition Requirements

Auxiliary test voltage was injected at frequencies of 80 Hz, 125.5 Hz, 176 Hz, and 200 Hz, and amplitudes of 29 V, 36 V, 43 V, and 46.5 V, respectively. To inject the test frequency, different options have been tested, including the use of a synchronous machine to achieve a complete sinusoidal auxiliary supply. At present, an AC frequency inverter is used which is able to inject an auxiliary test voltage from 0 Hz to 400 Hz and from 0 to 250 VAC.

Frequency sidebands were checked around some of the new current harmonics obtained in (10), especially:

$$f_{c1}=-2f_{s}+f_{i},\;\;\;f_{c2}=2f_{s}+f_{i},\;\;\;f_{c3}=f_{s}+2f_{i}$$

where *f_ci_* is the composed frequency (Table 1). New fault harmonics are expected at frequencies provided by (10).

Several tests have been carried out taking the aforementioned into account. These validate the idea of using an auxiliary voltage test signal and analyzing the sideband harmonics for the detection of a faulty induction motor.

The load was adjusted by means of a DC motor working as a generator and by supplying a set of resistors. The motor was supplied with 220 VAC, star connection. This means 150 V AC per phase, which leads to a speed lower than the nominal (1,275 rpm), and a slip frequency higher than the nominal value (approximately 15%). Using this connection does not affect the main conclusions of the paper, although the results are shown in a much clearer manner.

**Figure 14.**
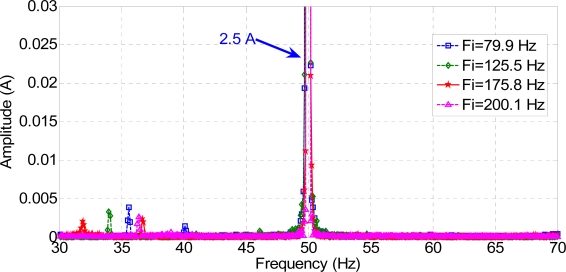
Stator current spectrum for a healthy motor with a load.

**Figure 15.**
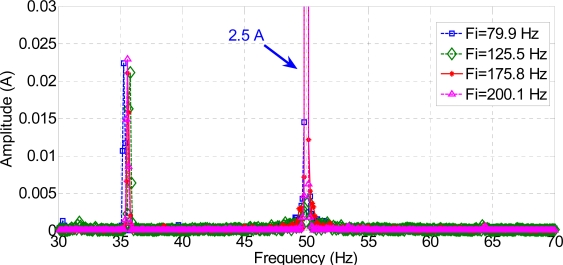
Stator current spectrum for a faulty motor with a load.

Figures 14 and 15 show the standard MCSA spectrum around the main frequency of 50 Hz, both for a healthy and for a faulty motor, and for each frequency injected. The rotor was running at 1,275 rpm, and the faulty frequencies for broken rotor bars are shown at 15 Hz from the generating frequency, approximately (Figure 14). The ratio between the harmonic due to the fault and the main harmonic is lower than 1%. This result agrees with that expected from applying the classical MCSA method.

**Figure 16.**
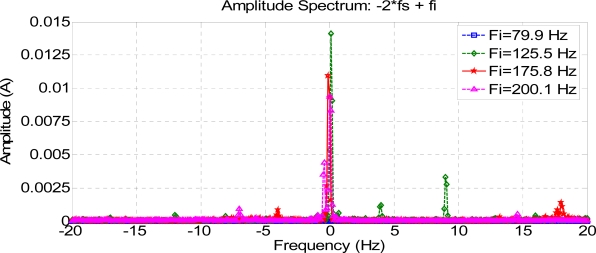
Stator current spectrum around fc1 for a healthy motor.

**Figure 17.**
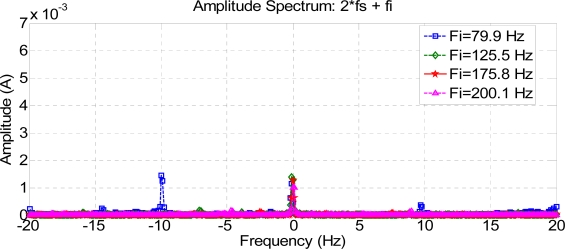
Stator current spectrum around fc2 for a healthy motor.

**Figure 18.**
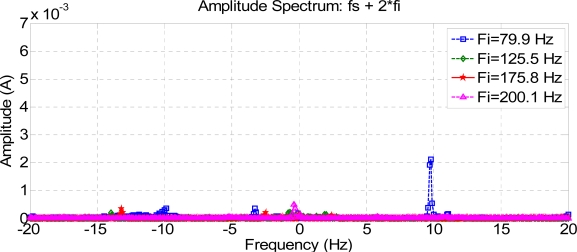
Stator current spectrum around fc3 for a healthy motor.

The current spectra around fc1, fc2, and fc3 for every frequency injected, for a healthy motor, are shown in Figures 16, 17 and 18. To show the effects of every frequency better, composition frequencies were centered at 0 Hz and the resulting faulty frequencies were located around this central position.

As expected, frequency compositions fc1 have higher amplitude than fc2 and fc3 in a healthy motor, because they are at a greater distance from the pole of the low-pass motor filter.

**Figure 19.**
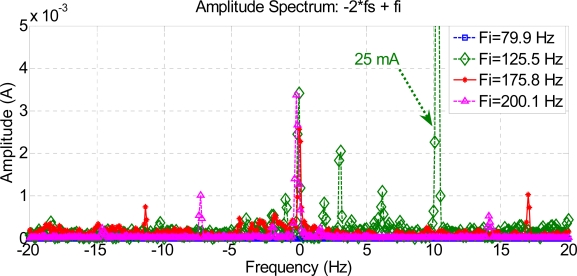
Stator current spectrum around fc1 for a faulty motor.

**Figure 20.**
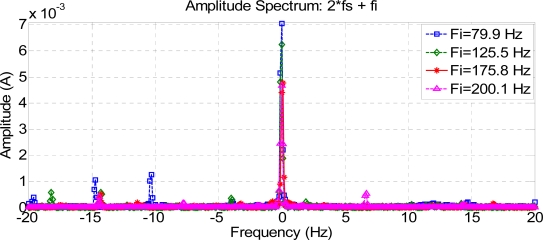
Stator current spectrum around fc2 for a faulty motor.

**Figure 21.**
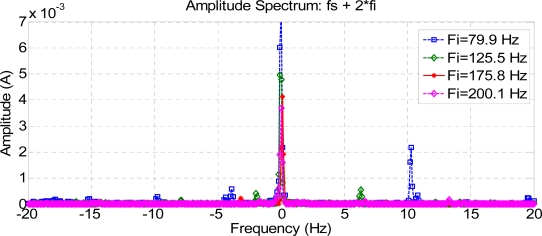
Stator current spectrum around fc3 for a faulty motor.

Figures 19, 20 and 21 show the current spectrum around fc1, fc2, and fc3 for every frequency injected to a faulty motor. As expected, the corresponding current spectrum component due to the fault is −15 Hz in every figure. However, the spectrum around fc1 has plenty of different harmonics, which makes it difficult to identify the fault. This is because the centered frequencies are 25.5 Hz, 76 Hz and 100 Hz, and the sidebands are in the range of 5 Hz to 120 Hz. It is in this range that we can locate most harmonics in a real machine: rotor eccentricities, flux unbalances, and mechanical shocks, among others. On the other hand, Figure 20 and Figure 21 show much clearer spectra, although the amplitudes of the harmonics are lower around fc3 because they are close to the cut-off frequency of the low-pass motor filter.

Although the amplitude of these new fault components is quite reduced, the 10% ratio found between the fault frequency and the generating frequency is higher than the 1% ratio calculated for the standard components used in the classical MCSA (Figure 12).

Generating frequencies in Figure 19 are of the same order as the main frequency. This means that the test signals affect the motor’s operation, to then change the slip. This fact, combined with the unclear spectrum, makes low-frequency compositions fc1 unsuitable for the detection of rotor faults.

Figure 20 and Figure 21 show faulty frequencies exactly with the expected values. However, the generating frequencies are too large in the case of fc3 and the resulting harmonics are too small and difficult to measure and analyze. On the contrary, Figure 20 shows not only an excellent relationship between generating and resulting frequencies of about 11%, but also a fault harmonic amplitude of 2 e-3A, which is enough to be obtained and analyzed. Therefore, the proposed method consists of capturing and analyzing these new current spectral components that appear due to the signal composition between main and injected frequencies.

Some relatively important harmonics appear in the spectra for both healthy and faulty machines. For instance, Figure 17 and Figure 20 show a −10 Hz frequency component of 1.5e-3 A for Fi = 80 Hz, which corresponds to 170 Hz in the stator current spectrum. This component, which is not directly related to the fault, is due to the frequency composition (5Fs–Fi). A similar explanation can be offered for the +10 Hz frequency component of 2e-3 A in Figures 18 and 21, which is due to the frequency composition (3Fs–Fi). In this case, the real stator component is 220 Hz. Obviously, all these frequencies which are due to frequency compositions given by (10) should not be considered for fault analysis.

**Figure 22.**
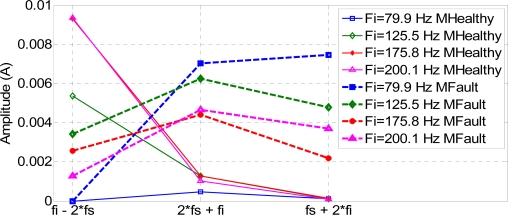
Amplitude of the stator composed frequencies.

The amplitude of the compound frequencies fci in the stator current spectrum is shown in Figure 22. From the figure, it can be concluded that the magnitude of fc1 in a healthy motor is larger than in a faulty motor. However, the magnitude of fc2 and fc3 in a healthy motor is smaller than for a faulty motor.

These conclusions are applicable to every frequency injected. Thus, specific compositions fc2 and fc3 could also be used to detect rotor failures, because their amplitude, for every frequency injected, is clearly higher in the damaged motor.

To detect a fault, the sideband around the expected fault frequency is monitored for a period of time after applying a test frequency. The diagnostic system will look for a specific harmonic amplitude increase. If it appears, and the relationship between the generating frequency fc2 and the fault frequency is higher than a predetermined value, then the fault will be detected. Compared with the standard MCSA method, the only drawback is that it is necessary to generate and apply the test signal to the stator phases. However, the generation of a 75–200 Hz sine wave is not a problem for the modulator included in every present frequency inverter. On the other hand, the measurement of the current phases is already used in the MCSA method, as well as for control purposes.

The selection of the test signal frequency is a trade-off between several concerns. The carrier frequency must be high enough to create a deep bar effect that prevents the high frequency flux wave from substantially linking to the rotor bars, but it must also be low enough so that the skin effect in the rotor laminations does not repel the flux from penetrating below the rotor surface.

In a practical case, a low-pass filter model of the machine can be proposed, with the pole frequency in 400 Hz. Therefore, the interaction between main and signal test frequencies should cause new harmonic components lower than this value in order to get good results.

In case of incipient fault condition the appearance of fault harmonics and composed harmonics remains. However, the amplitude of harmonics is directly related with the fault condition. other testing has also been carried out with inverter supply and low fault condition 1 and 2 BRB. In the following it is shown and the main testing results are discussed

**Figure 23.**
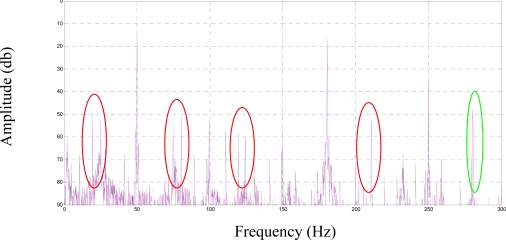
Band Current Spectrum for 1 BRB motor.

Main Supply, Vphase = 230 Vrms    f = 50 Hz
Test voltage, Vphase =20 Vrms   f_1_ = 80 Hz, f_2_ = 125 Hz;

Figure 23 shows the fault condition and the compositions off signals over the spectrum.

### 4.3. Mention for VVVF Converter Supply

**Figure 24.**
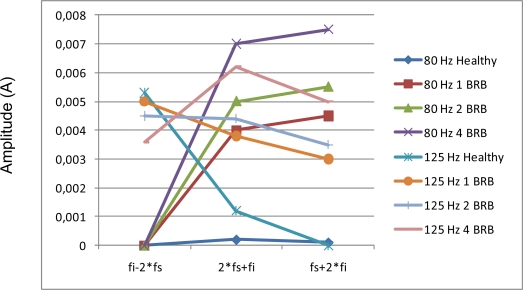
Amplitude comparison of the stator composed frequencies for different fault condition.

Although the injected voltage was obtained from an auxiliary generator through a serial transformer, there is no problem to generate a composed three-phase sine wave with the desired test frequency by using a special modulation reference in the Space Vector Modulation block of the power inverter. For a practical implementation in industrial equipment, the frequency test signal should be higher than the bandwidth of the current loop, especially when vectorial control is applied to IM. In that case, the choice of frequency test signal will be the same as in sinusoidal application, more or less on the 80–200 Hz band. In order to allow subharmonics due to the modulation we introduce a reactance high-pass filter between the drive and the VVVF converter, which cuts subharmonics due to an asynchronous modulation. Figure 24 shows the amplitude comparison between composed harmonics for 1 Broken Rotor Bar, 2 and 4. The injected frequencies chosen have been the most promising ones for fault detection (80 Hz and 125 Hz).

## 5. Conclusions

Signal injection ensures proper results in the detection of faults, especially in cases of low torque. The use of an anti-clockwise injected frequency introduces additional slip on the motor which allows the detection of faults with a better dynamic resolution. Furthermore, the composed frequencies are good indicators of the behavior of machine faults. It has been clearly demonstrated that in the case of a fault condition some of these composed frequencies increase their values, which implies unbalances in the machine that could be understood as a fault condition.

However, the composed frequencies only introduce the notion of unbalances, but they cannot differentiate between rotor misalignments and BRB fault conditions, in order to get a proper diagnosis. The fault condition could be distinguished by analyzing the current spectral distribution about injected and composed harmonics, but the location of faulty harmonics depends on the slip value, which means that in case of a variable load the fault condition cannot be clearly appreciated.

In conclusion, it is possible to establish that:

– The signal injection technique is a good method for fault detection under low load, through examination of the fault harmonics on the injected signal and the frequency compositions.
– The signal injection technique is a good estimator of conditions of unbalance, through examination of the amplitude of the composed frequency.
– In case of a variable load, the composed frequency should ensure unbalance, but improvements will be needed in the field of signal processing to distinguish fault conditions.

## Figures and Tables

**Table 1.** Injected and Composed Frequencies.

Supply Frequency (*f_s_*) = 50 Hz			

Injected Frequency (*f_i_*) Hz	*f_c1_* = −*2f_s_* + *f_i_* Hz	*f_c2_* = *2f_s_* + *f_i_* Hz	*f_c3_* = *f_s_* + *2f_i_* Hz
79.9	−20.1	179.9	209.8
125.5	25.5	225.5	301
175.8	75.8	275.8	401.6
200	100	300	450
